# Supplementary figures and images for: Phagocytosed Polyhedrin-Cytokine Cocrystal Nanoparticles Provide Sustained Secretion of Bioactive Cytokines from Macrophages
Source: Biodes Res. 2021 May 14;2021:9816485. doi: 10.34133/2021/9816485 (PMC10521757; doi:10.34133/2021/9816485)

# Figure S1

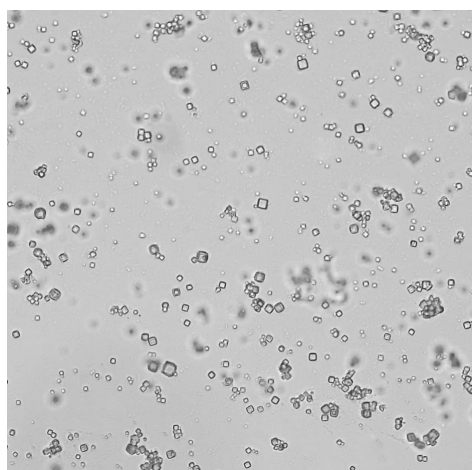

Brightfield 40x

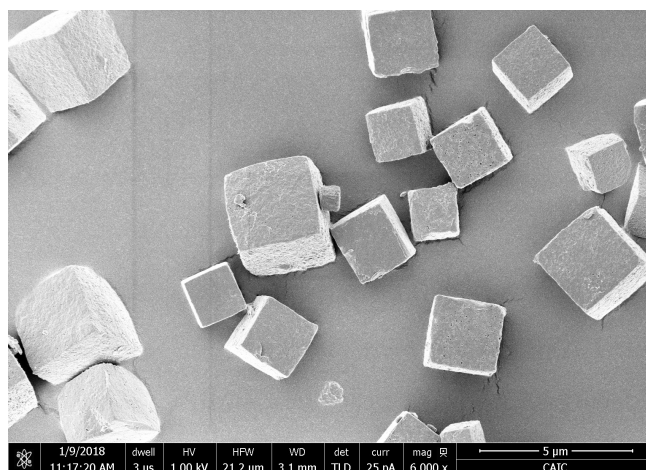

SEM

Supplement: Supplementary 1 — Supp fig 1: PODS morphology. PODS Empty was spun down onto a tissue culture plastic and imaged using SEM (left image) and brightfield microscopy (right image) at a magnification of 40x. PODS are easily distinguishable from cells due to their cubic shape. [file 9816485.f1.pdf]

# Figure S2

Frame 59

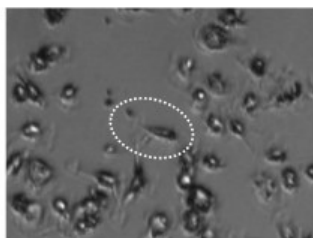

Frame 70

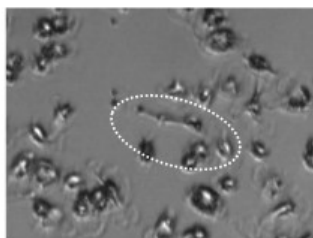

Frame 76

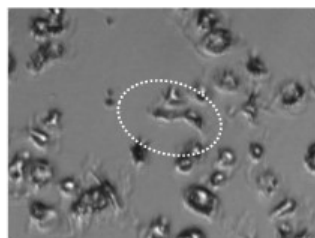

Frame 80

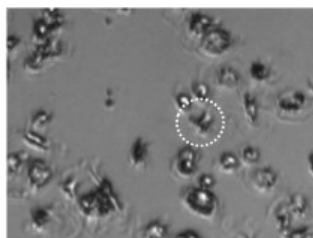

Supplement: Supplementary 3 — Supp fig 2: GM-CSF-loaded mouse BMDMs were incubated with PODS eGFP for 24 h and monitored using a live cell imaging system at 1 frame/min. The dashed white circle follows the movement of a macrophage towards a single PODS, the uptake of the PODS, and the contraction of the macrophage over 21 min depicted in a still of 4 frames (59, 70, 76, and 80). [file 9816485.f3.pdf]

# Figure S3

Frame 1

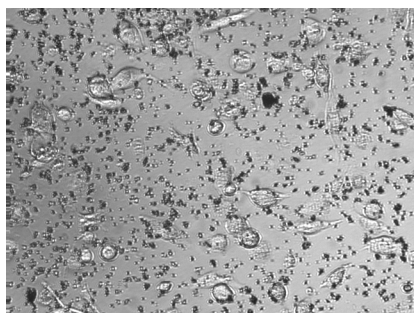

Frame 675

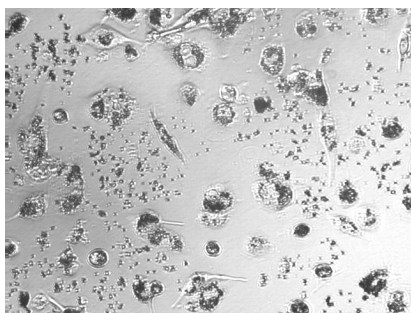

Frame 1335

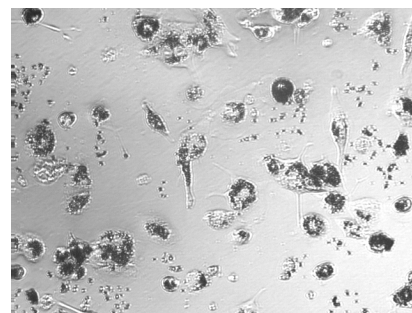

Supplement: Supplementary 5 — Supp Fig 3: efficient uptake of PODS into M0. THP-1 cells were differentiated first into M0 macrophages and then further polarized into M1 cells. Uptake of PODS Empty was allowed for 24 h and monitored using a live cell imaging system at 1 frame/min. Three frames (1, 675, and 1335) are shown to illustrate the uptake over time. [file 9816485.f5.pdf]

Figure S4

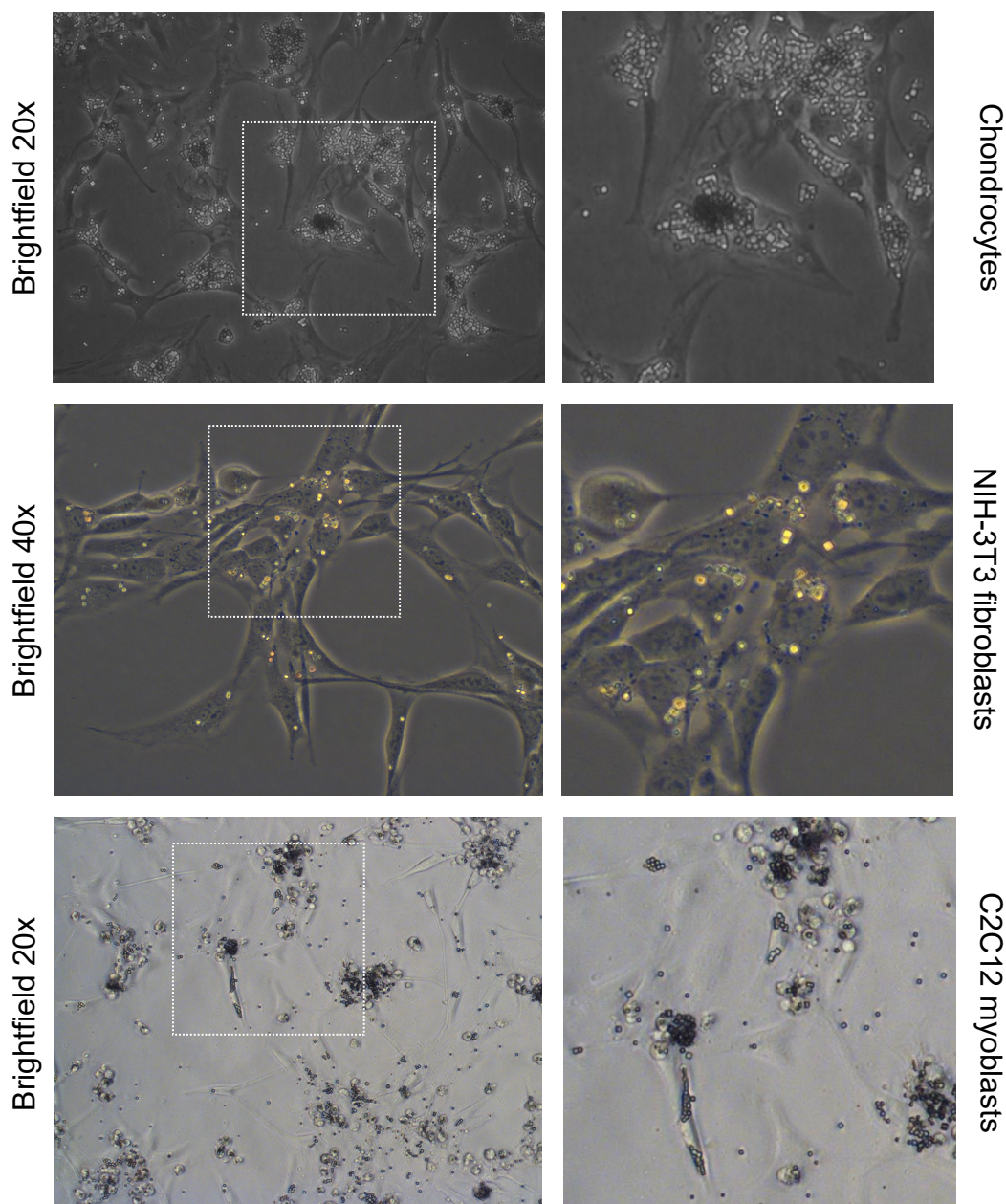

Supplement: Supplementary 6 — Supp Fig 4: phagocytosis of PODS protein crystals into nonprofessional phagocytes. Chondrocytes, NIH3T3 cells, and C2C12 cells were cultured with PODS Empty for 24 h and subsequently imaged with brightfield microscopy at either 20x or 40x magnification. The dashed square marks the area of the zoom shown in the right image of each panel. [file 9816485.f6.pdf]

# Figure S5

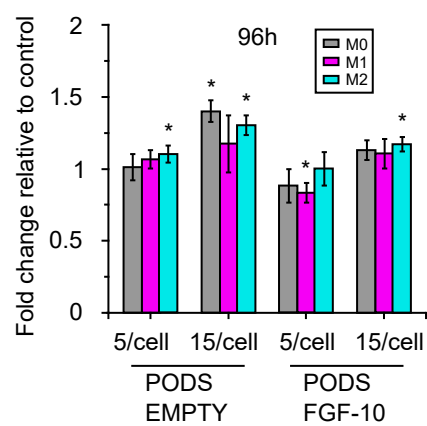

Supplement: Supplementary 7 — Supp Fig 5: viability of PODS-loaded macrophages after 96 h. M0, M1, and M2 macrophages were incubated with PODS Empty or PODS FGF-10 (1 : 5 and 1 : 15) for 24 h in a 96-well TC plate. Subsequently, the medium was changed and viability of cells was measured 96 h after PODS uptake using a colorimetric assay (Orangu™, Cell Guidance Systems). The fold change in viability was calculated relative to unloaded macrophages of the same polarization state. Error bars represent the standard deviation of samples (n=3). ∗Significant differences to control (p<0.05). [file 9816485.f7.pdf]

# Figure S6

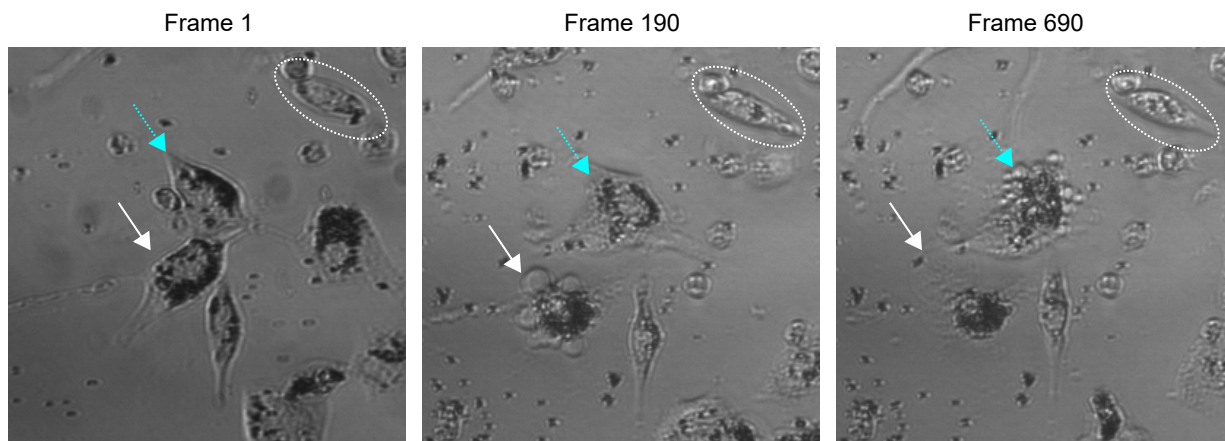

Supplement: Supplementary 8 — Supp Fig 6: overloading of macrophages leads to apoptosis. M1 cells already loaded with an average of 10 PODS per cell and were incubated with another 10 PODS per cell for further 24 h, and images were taken every 2 min. Three frames were chosen to follow 3 cells. The dashed white circle marks a healthy cell with approximately 10 PODS ingested. The white arrow and turquois arrow mark two cells with more than 50 PODS ingested; both cells undergo apoptosis shown in frame 190 and frame 690, respectively. [file 9816485.f8.pdf]
